# Retraction Note: Myricetin ameliorates ox-LDL-induced HUVECs apoptosis and inflammation via lncRNA GAS5 upregulating the expression of miR-29a-3p

**DOI:** 10.1038/s41598-026-69704-y

**Published:** 2026-09-09

**Authors:** Yunpeng Bai, Xiankun Liu, Qingliang Chen, Tongyun Chen, Nan Jiang, Zhigang Guo

**Affiliations:** 1 Chest hospital, Tianjin university, Tianjin, 300222 China; 2 Tianjin chest hospital, Tianjin medical university, Tianjin, 300222 China; 3 Graduate School, Tianjin Medical University, Tianjin, 300070 P. R. China

Retraction of: *Scientific Reports* https://doi.org/10.1038/s41598-021-98916-7, published online 04 October 2021.

The Editors have retracted this article.

After publication, concerns were raised regarding the data presented in the Figures, specifically:

- Fig. 3D ox-LDL + Myr and ox-LDL + Myr + pcDNA plots appear highly similar;
- Fig. 5D ox-LDL + Myr + pcDNA and ox-LDL + Myr + pcDNA-GAS5 + miR-29a-3p mimics plots appear highly similar;
- Fig. 6G ox-LDL + NC and ox-LDL + mimic control plots appear highly similar;
- Fig. 7 IkBa blot has eight lanes, although only seven groups were analysed in the presented experiment.

The Authors have stated that the errors in the flow cytometry plots occured due to duplicated FCS files, which affected subsequent analysis. The Editors therefore no longer have confidence in the presented data.

The Authors do not agree with this retraction.

